# The Renaissance of Developmental Biology

**DOI:** 10.1371/journal.pbio.1002149

**Published:** 2015-05-06

**Authors:** Daniel St Johnston

**Affiliations:** The Gurdon Institute and The Department of Genetics, University of Cambridge, Cambridge, United Kingdom

## Abstract

Since its heyday in the 1980s and 90s, the field of developmental biology has gone into decline; in part because it has been eclipsed by the rise of genomics and stem cell biology, and in part because it has seemed less pertinent in an era with so much focus on translational impact. In this essay, I argue that recent progress in genome-wide analyses and stem cell research, coupled with technological advances in imaging and genome editing, have created the conditions for the renaissance of a new wave of developmental biology with greater translational relevance.

## Abstract 2

A leader in the field explores why developmental biology has suffered from a relative decline in impact in recent years and presents a personal view as to why the time is ripe for its re-emergence as a key area of research.

*This Essay is part of the "Where Next*?*" Series*.

It is a commonly held view that the mid 1980s to 2000 represented the golden age of developmental biology. After all, it was during this period that genetic screens in worms and flies led to the discovery of the Notch [1,2], Decapentaplegic (Dpp), or bone morphogenetic protein (BMP) [3], Toll [4,5], Hedgehog [6,7], and Wingless (Wnt) [8,9] signalling pathways, and it was soon realized that these are conserved and play key roles in mammalian development and disease. Furthermore, the developmental genetics of this era led to the identification of the homeobox [10–12], the colinearity of the Hox genes in invertebrates and vertebrates [13,14], and several “master regulators” of organ or cellular identity [15,16]. Since the publication of the human genome sequence [17,18], however, the field of genomics and associated genome-wide approaches has become the hot area of research, largely eclipsing developmental biology. This change is reflected in the gradually declining impact factors of all developmental biology journals and the corresponding rise of “omics” journals [19]. Developmental biology has been further pushed to the sidelines by the growth of the field of stem cell biology following the derivation of induced pluripotent stem cells from mouse and human adult somatic cells [20,21] and the promise of rapid advances in regenerative medicine that these breakthroughs heralded. Indeed, it has almost become unfashionable to say that one is a developmental biologist, and I have been passing myself off as an “in vivo cell biologist” for a number of years. Does this mean that developmental biology is facing a continuing and inevitable decline in impact? In this essay, I contend that the opposite is the case and that, largely because of the recent advances in genomics and stem cell biology, we should look forward to the renaissance of developmental biology.

Although “omics” papers now occupy more space in the top journals than developmental biology ones, this field has also contributed enormously to our understanding of developmental mechanisms, particularly at the transcriptional level. Firstly, complete genome sequences have made it possible to knock down every gene in the *Caenorhabditis elegans* or *Drosophila* genomes in vivo using genome-wide RNAi libraries, which is a very effective way to screen for interesting developmental phenotypes [22,23]. Secondly, advances in Chip-Seq, RNA-seq, and chromosome conformation capture techniques are providing an unprecedented view of how transcription is controlled during development [24–29]. Until recently, these approaches have been largely limited to the analysis of populations of cells, but recent advances in single cell transcriptomics are beginning to extend these techniques to specific cell types and even individual cells within developing organisms [30–32]. These approaches can therefore start to address major unanswered questions into development. For instance, whole genome sequencing of many individual cells can reveal the inheritance patterns of random somatic mutations, from which one can infer lineage relationships and construct fate maps, which are difficult to determine by other means in mammalian embryos [33,34]. Perhaps even more significant is the use of single cell RNA-seq to classify cell types on the basis of their transcriptional profiles [35]. Although the literature contains the assertion that there are about 210 distinct cell types in humans, this is almost certainly too low by at least an order of magnitude. Single cell transcriptional profiling has the potential to reveal the full repertoire of cell types that compose our bodies, which is an essential prerequisite for understanding how it is constructed.

Given the rapid advances in genomic technologies, it is conceivable that we will soon have a detailed picture of the genome-wide distribution of chromatin states, transcription factor binding site occupancy, and mRNA and noncoding RNA transcriptomes for most cells in an organism. This will provide a wealth of information about how the epigenetic landscape interacts with tissue-specific transcription factors to control cell fates. Important though this is, however, the net output will be an inventory of which genes are expressed where and when in the developing animal. Just as mapping every synapse in the human brain is unlikely to reveal the basis of consciousness, knowing the complement of expressed genes in every cell will not explain how a tissue, organ, or whole organism acquires its form and function. Interpreting this large amount of data will require understanding how this intrinsic information is integrated with external signals and cues to control cell behaviour, a topic that lies at the heart of developmental biology. In other words, the “omics” revolution can provide the raw material for generating interesting hypotheses about how animals develop, but a great deal of developmental biology research will be needed to work out how the linear information of the genome and transcriptome is transformed into a three-dimensional cellular structure. Amongst other things, this will necessitate understanding how cell shape and cell movement are controlled in different contexts, how the direction and range of cell signalling are regulated, how cells produce and are influenced by mechanical forces, and how the collective behaviours of groups of cells reproducibly generate complex structures.

The field of stem cell biology is probably even hotter than that of genomics, with many countries targeting significant proportions of their research budgets towards this strategic area. Indeed, a third of this year’s applications for European Research Council Starting Grants in Cell and Developmental Biology were on stem cell-related topics. Stem cells are central to developmental biology, since they are the founder cells of most, if not all, developmental lineages. At present, however, most stem cell research is more applied and uses tissue culture assays to investigate questions such as the nature of “stemness” or which factors can induce pluripotent stem cells to differentiate into specific cell types. This has caused heated discussion in the developmental biology community about what its relationship should be to the stem cell field. For example, the British Society of Developmental Biology recently held its longest general meeting for years to consider whether we should change our name to the British Society of Developmental Biology and Stem Cells, a motion that was narrowly defeated after much debate [36].

This existentialist angst is partly driven by a desire to be associated with a well-funded and exciting field but also reflects the profound links between developmental biology and stem cell research. Takahashi and Yamanaka [21] did not pick transcription factors at random when trying to induce pluripotent stem cells, as two of the four factors, Oct4 and Sox2, were chosen because previous work in the early mouse embryo had shown that they are required for the pluripotency of inner cell mass cells [37,38]. One of the most remarkable recent advances in the stem cell field has been the development of multistep protocols that use scores of factors and inhibitors to induce embryonic or induced pluripotent stem cells to differentiate into specific cell types, most notably insulin-secreting pancreatic β-cells [39,40]. Many steps in these extremely complicated protocols are based on understanding and recapitulating the normal development of the pancreas, including in vivo studies that identified the signalling factors that induce endoderm, then foregut, then pancreatic endoderm, and finally endocrine precursor cells [41–46]. Finally, in vivo studies are beginning to reveal that the special properties of stem cells are not so unique, and that differentiated cells can de-differentiate and regain pluripotency. For instance, multicellular germline cysts in *Drosophila* and mice can fragment, de-differentiate, and become germline stem cells [47–49], while ductal cells of the liver and even newt muscle syncytia can revert to a stem cell state in response to damage [50,51].

For me, one of the most exciting aspects of the recent explosion of stem cell research is the amazing amount of self-organisation that can take place in vitro under appropriate culture conditions. No one would ever have predicted that a uniform starting population of embryonic stem (ES) cells could spontaneously undergo morphogenesis in culture to give rise to an optic cup with a stratified neural retina [52], that ES or induced pluripotent stem (iPS) cells could be cultured to form cerebral organoids, or “mini brains,” with defined cortical regions [53], or that a single Lgr5^+^ intestinal cell could form an intestinal epithelium with crypts and villi [54,55] (Fig 1). The number of organ-like structures that can now be grown in tissue culture is increasing exponentially and now includes most regions of the gut [55–58], liver [50], pancreas [59,60], salivary gland [61], skin[62], prostate gland[63], Rathke’s pouch [64], neural tube [65], lung[66], and even embryoid bodies that break symmetry and recapitulate some of the cell movements of gastrulation [67]. Although it will be important to confirm that these systems recapitulate normal development, the complexity of the structures produced and their similarities to the real in vivo organs suggest this is likely to be the case. They therefore provide fantastic models for studying mammalian and, particularly, human development.

**Fig 1:**
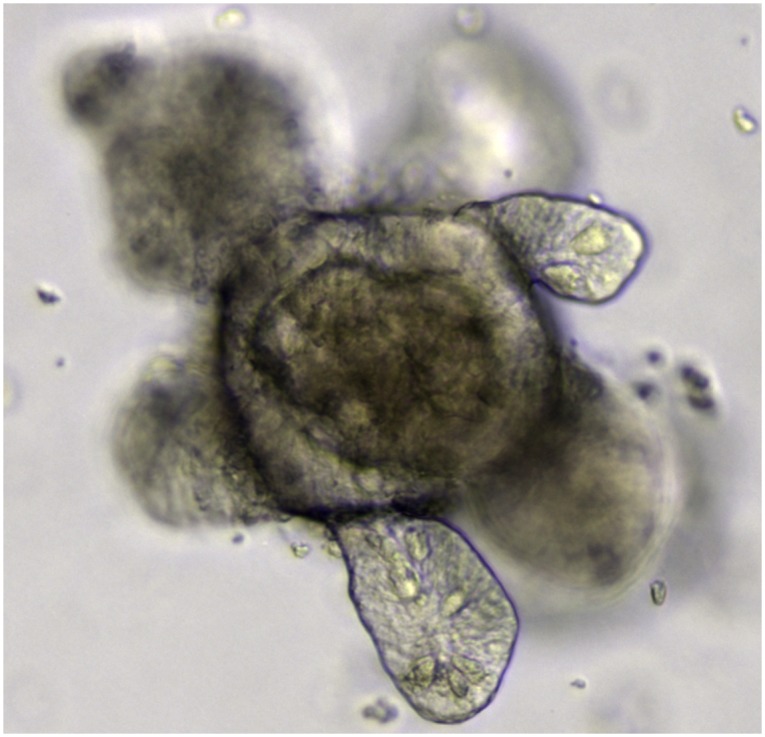
An intestinal organoid grown from Lgr5^+^ stem cells (courtesy of Meritxell Huch).

The study of human development has been hampered by the inaccessibility of the embryo in utero inside the mother and by the obvious ethical rules against performing experiments on foetuses. Both of these problems are overcome by organoid systems, several of which can be grown from iPS cells [66, 68–70]. We are therefore now in a position to study how normal and diseased human tissues develop in vitro. This means that they can be observed by time-lapse imaging and analysed with the whole array of sophisticated tools that have been so successful in model organisms. A better understanding of the mechanisms that allow organoids to self-organise and undergo morphogenesis should also inform more translational research into regenerative medicine and disease modelling. After all, one wouldn’t want to use a technology on oneself without understanding how and why the components work.

An additional reason for the relative decline in the popularity of developmental biology in recent years has been the mounting pressure in many countries to focus on translational research, as this increasingly means research on humans, whereas most research on animal development until now has been carried out using worms, flies, fish, frogs, and mice. Studying the development of human organoids therefore provides a way for the field to meet this translational agenda. This does not mean we should stop research on the classic model organisms: they still provide the most amenable systems for understanding many key developmental processes and are the only systems where one can examine the development of the whole organism or the relationship between development and physiology. Nevertheless, many insights in development have come from comparisons between organisms and using each system for the experiments to which it is most suited. The addition of organoids to the developmental biologist’s repertoire will not only provide “human relevance” but will inform and enhance our understanding of developmental processes.

Both the “omics” and stem cell revolutions have been based on technological advances: for the former, the development of high throughput sequencing [71,72], and for the latter, the discovery of the Yamanaka factors [20,21,73] that proved that cellular reprogramming is possible. Developmental biology has not undergone such a dramatic revolution in recent years, perhaps because a wider diversity of techniques and approaches are needed to investigate how cells and groups of cells change fate, shape, and position during the formation of complex structures. Nevertheless, two recent advances are likely to enormously enhance our ability to analyse the complexity of development. Firstly, new microscopy techniques, such as light sheet, imaging make it possible to perform live imaging of thick specimens, such as embryos or organoids, at video rates in three dimensions [74–76], while advances in super-resolution microscopy will soon allow the observation of developmental processes with molecular precision in real time [77–80]. Secondly, the recent adaptation of clustered regularly interspaced palindromic repeat (CRISPR)/CAS9 technology to eukaryotes promises to transform many aspects of the field [81,82]. CRISPR can generate gene knock-outs in almost any organism or cultured cell line and can even be used to perform genome-wide screens in tissue culture using lentiviral libraries [83–85]. Furthermore, because the double-strand DNA breaks induced by the Cas9 nuclease/guide RNA complex stimulate homologous recombination, one can easily introduce specific mutations or fluorescent tags into a gene of interest by providing appropriate repair templates [86,87]. This will allow sophisticated imaging and genetic analysis in nonstandard organisms and organoid systems. We therefore have an increasingly effective collection of tools to answer the exciting questions that recent advances in stem cell and “omics” research have raised.

The field of solid state physics was revitalized a few years ago by changing its name to condensed matter physics, and it may be that the developmental biology would benefit from rebranding itself in much the same way, for example, by becoming development and regenerative medicine, or four dimensional biology, or something even more catchy. Nevertheless, whatever name the field settles on, I predict that the power of these new approaches and the significance of the problems still to be solved will make the next decade a golden age for developmental biology.

## Abbreviations

BMP: bone morphogenetic protein
CRISPR: clustered regularly interspaced palindromic repeat
Dpp: Decapentaplegic
ES: embryonic stem
iPS: induced pluripotent stem
Wnt: Wingless

## References

[1] Artavanis-Tsakonas S, Muskavitch MA, Yedvobnick B (1983) Molecular cloning of Notch, a locus affecting neurogenesis in Drosophila melanogaster. Proc Natl Acad Sci U S A 80: 1977–1981. 6403942 10.1073/pnas.80.7.1977 PMC393735

[2] Kopczynski CC, Alton AK, Fechtel K, Kooh PJ, Muskavitch MAT (1988) Delta, a Drosophila Neurogenic Gene, Is Transcriptionally Complex and Encodes a Protein Related to Blood-Coagulation Factors and Epidermal Growth-Factor of Vertebrates. Genes & Development 2: 1723–1735. 3149249 10.1101/gad.2.12b.1723

[3] Padgett RW, St Johnston RD, Gelbart WM (1987) A transcript from a Drosophila pattern gene predicts a protein homologous to the transforming growth factor-beta family. Nature 325: 81–84. 3467201 10.1038/325081a0

[4] Hashimoto C, Hudson KL, Anderson KV (1988) The Toll Gene of Drosophila, Required for Dorsal-Ventral Embryonic Polarity, Appears to Encode a Transmembrane Protein. Cell 52: 269–279. 2449285 10.1016/0092-8674(88)90516-8

[5] Anderson KV, Jurgens G, Nussleinvolhard C (1985) Establishment of Dorsal-Ventral Polarity in the Drosophila Embryo—Genetic-Studies on the Role of the Toll Gene-Product. Cell 42: 779–789. 3931918 10.1016/0092-8674(85)90274-0

[6] Lee JJ, Vonkessler DP, Parks S, Beachy PA (1992) Secretion and Localized Transcription Suggest a Role in Positional Signaling for Products of the Segmentation Gene Hedgehog. Cell 71: 33–50. 1394430 10.1016/0092-8674(92)90264-d

[7] Tabata T, Eaton S, Kornberg TB (1992) The Drosophila Hedgehog Gene Is Expressed Specifically in Posterior Compartment Cells and Is a Target of Engrailed Regulation. Genes & Development 6: 2635–2645. 1340474 10.1101/gad.6.12b.2635

[8] Baker NE (1987) Molecular cloning of sequences from wingless, a segment polarity gene in Drosophila: the spatial distribution of a transcript in embryos. EMBO J 6: 1765–1773. 16453776 10.1002/j.1460-2075.1987.tb02429.x PMC553553

[9] Rijsewijk F, Schuermann M, Wagenaar E, Parren P, Weigel D, et al (1987) The Drosophila homolog of the mouse mammary oncogene int-1 is identical to the segment polarity gene wingless. Cell 50: 649–657. 3111720 10.1016/0092-8674(87)90038-9

[10] Mcginnis W, Garber RL, Wirz J, Kuroiwa A, Gehring WJ (1984) A Homologous Protein-Coding Sequence in Drosophila Homeotic Genes and Its Conservation in Other Metazoans. Cell 37: 403–408. 6327065 10.1016/0092-8674(84)90370-2

[11] Mcginnis W, Levine MS, Hafen E, Kuroiwa A, Gehring WJ (1984) A Conserved DNA-Sequence in Homoeotic Genes of the Drosophila Antennapedia and Bithorax Complexes. Nature 308: 428–433. 6323992 10.1038/308428a0

[12] Scott MP, Weiner AJ (1984) Structural Relationships among Genes That Control Development—Sequence Homology between the Antennapedia, Ultrabithorax, and Fushi Tarazu Loci of Drosophila. Proceedings of the National Academy of Sciences of the United States of America-Biological Sciences 81: 4115–4119. 6330741 10.1073/pnas.81.13.4115 PMC345379

[13] Krumlauf R (1992) Evolution of the Vertebrate Hox Homeobox Genes. Bioessays 14: 245–252. 1350721 10.1002/bies.950140408

[14] Duboule D, Morata G (1994) Colinearity and Functional Hierarchy among Genes of the Homeotic Complexes. Trends in Genetics 10: 358–364. 7985240 10.1016/0168-9525(94)90132-5

[15] Halder G, Callaerts P, Gehring WJ (1995) Induction of ectopic eyes by targeted expression of the eyeless gene in Drosophila. Science 267: 1788–1792. 7892602 10.1126/science.7892602

[16] Weintraub H, Tapscott SJ, Davis RL, Thayer MJ, Adam MA, et al (1989) Activation of muscle-specific genes in pigment, nerve, fat, liver, and fibroblast cell lines by forced expression of MyoD. Proc Natl Acad Sci U S A 86: 5434–5438. 2748593 10.1073/pnas.86.14.5434 PMC297637

[17] Lander ES, Consortium IHGS, Linton LM, Birren B, Nusbaum C, et al (2001) Initial sequencing and analysis of the human genome. Nature 409: 860–921. 11237011 10.1038/35057062

[18] Venter JC (2001) The sequence of the human genome (vol 292, pg 1304, 2001). Science 292: 1838–1838.

[19] Journal Citation Reports (2014), Thomson Reuters.

[20] Takahashi K, Tanabe K, Ohnuki M, Narita M, Ichisaka T, et al (2007) Induction of pluripotent stem cells from adult human fibroblasts by defined factors. Cell 131: 861–872. 18035408 10.1016/j.cell.2007.11.019

[21] Takahashi K, Yamanaka S (2006) Induction of pluripotent stem cells from mouse embryonic and adult fibroblast cultures by defined factors. Cell 126: 663–676. 16904174 10.1016/j.cell.2006.07.024

[22] Dietzl G, Chen D, Schnorrer F, Su KC, Barinova Y, et al (2007) A genome-wide transgenic RNAi library for conditional gene inactivation in Drosophila. Nature 448: 151–156. 17625558 10.1038/nature05954

[23] Kamath RS, Fraser AG, Dong Y, Poulin G, Durbin R, et al (2003) Systematic functional analysis of the Caenorhabditis elegans genome using RNAi. Nature 421: 231–237. 12529635 10.1038/nature01278

[24] Gerstein MB, Rozowsky J, Yan KK, Wang D, Cheng C, et al (2014) Comparative analysis of the transcriptome across distant species. Nature 512: 445–448. 10.1038/nature13424 25164755 PMC4155737

[25] Ho JW, Jung YL, Liu T, Alver BH, Lee S, et al (2014) Comparative analysis of metazoan chromatin organization. Nature 512: 449–452. 10.1038/nature13415 25164756 PMC4227084

[26] Negre N, Brown CD, Ma L, Bristow CA, Miller SW, et al (2011) A cis-regulatory map of the Drosophila genome. Nature 471: 527–531. 10.1038/nature09990 21430782 PMC3179250

[27] Lieberman-Aiden E, van Berkum NL, Williams L, Imakaev M, Ragoczy T, et al (2009) Comprehensive mapping of long-range interactions reveals folding principles of the human genome. Science 326: 289–293. 10.1126/science.1181369 19815776 PMC2858594

[28] van de Werken HJ, Landan G, Holwerda SJ, Hoichman M, Klous P, et al (2012) Robust 4C-seq data analysis to screen for regulatory DNA interactions. Nat Methods 9: 969–972. 10.1038/nmeth.2173 22961246

[29] Dixon JR, Selvaraj S, Yue F, Kim A, Li Y, et al (2012) Topological domains in mammalian genomes identified by analysis of chromatin interactions. Nature 485: 376–380. 10.1038/nature11082 22495300 PMC3356448

[30] Shapiro E, Biezuner T, Linnarsson S (2013) Single-cell sequencing-based technologies will revolutionize whole-organism science. Nat Rev Genet 14: 618–630. 10.1038/nrg3542 23897237

[31] Southall TD, Gold KS, Egger B, Davidson CM, Caygill EE, et al (2013) Cell-Type-Specific Profiling of Gene Expression and Chromatin Binding without Cell Isolation: Assaying RNA Pol II Occupancy in Neural Stem Cells. Developmental Cell 26: 101–112. 10.1016/j.devcel.2013.05.020 23792147 PMC3714590

[32] van Steensel B, Henikoff S (2000) Identification of in vivo DNA targets of chromatin proteins using tethered Dam methyltransferase. Nature Biotechnology 18: 424–428. 10748524 10.1038/74487

[33] Carlson CA, Kas A, Kirkwood R, Hays LE, Preston BD, et al (2012) Decoding cell lineage from acquired mutations using arbitrary deep sequencing. Nature Methods 9: 78–U193. 10.1038/nmeth.1781 22120468 PMC3248619

[34] Frumkin D, Wasserstrom A, Kaplan S, Feige U, Shapiro E (2005) Genomic variability within an organism exposes its cell lineage tree. Plos Computational Biology 1: 382–394. 10.1371/journal.pcbi.0010050 PMC1274291 16261192

[35] Shapiro E, Biezuner T, Linnarsson S (2013) Single-cell sequencing-based technologies will revolutionize whole-organism science. Nature Reviews Genetics 14: 618–630. 10.1038/nrg3542 23897237

[36] Robertson E (2013) BSDB Chair's report. BSDB Newsletter pp. 2.

[37] Avilion AA, Nicolis SK, Pevny LH, Perez L, Vivian N, et al (2003) Multipotent cell lineages in early mouse development depend on SOX2 function. Genes Dev 17: 126–140. 12514105 10.1101/gad.224503 PMC195970

[38] Nichols J, Zevnik B, Anastassiadis K, Niwa H, Klewe-Nebenius D, et al (1998) Formation of pluripotent stem cells in the mammalian embryo depends on the POU transcription factor Oct4. Cell 95: 379–391. 9814708 10.1016/s0092-8674(00)81769-9

[39] Pagliuca FW, Millman JR, Gurtler M, Segel M, Van Dervort A, et al (2014) Generation of functional human pancreatic beta cells in vitro. Cell 159: 428–439. 10.1016/j.cell.2014.09.040 25303535 PMC4617632

[40] Rezania A, Bruin JE, Arora P, Rubin A, Batushansky I, et al (2014) Reversal of diabetes with insulin-producing cells derived in vitro from human pluripotent stem cells. Nat Biotechnol 32: 1121–1133. 10.1038/nbt.3033 25211370

[41] Apelqvist A, Li H, Sommer L, Beatus P, Anderson DJ, et al (1999) Notch signalling controls pancreatic cell differentiation. Nature 400: 877–881. 10476967 10.1038/23716

[42] Gamer LW, Wright CV (1995) Autonomous endodermal determination in Xenopus: regulation of expression of the pancreatic gene XlHbox 8. Dev Biol 171: 240–251. 7556900 10.1006/dbio.1995.1275

[43] Hebrok M, Kim SK, St Jacques B, McMahon AP, Melton DA (2000) Regulation of pancreas development by hedgehog signaling. Development 127: 4905–4913. 11044404 10.1242/dev.127.22.4905

[44] Kim SK, Hebrok M, Li E, Oh SP, Schrewe H, et al (2000) Activin receptor patterning of foregut organogenesis. Genes Dev 14: 1866–1871. 10921901 PMC316826

[45] Murtaugh LC, Stanger BZ, Kwan KM, Melton DA (2003) Notch signaling controls multiple steps of pancreatic differentiation. Proc Natl Acad Sci U S A 100: 14920–14925. 14657333 10.1073/pnas.2436557100 PMC299853

[46] Ninomiya H, Takahashi S, Tanegashima K, Yokota C, Asashima M (1999) Endoderm differentiation and inductive effect of activin-treated ectoderm in Xenopus. Dev Growth Differ 41: 391–400. 10466926 10.1046/j.1440-169x.1999.00449.x

[47] Brawley C, Matunis E (2004) Regeneration of male germline stem cells by spermatogonial dedifferentiation in vivo. Science 304: 1331–1334. 15143218 10.1126/science.1097676

[48] Kai T, Spradling A (2004) Differentiating germ cells can revert into functional stem cells in Drosophila melanogaster ovaries. Nature 428: 564–569. 15024390 10.1038/nature02436

[49] Nakagawa T, Sharma M, Nabeshima Y, Braun RE, Yoshida S (2010) Functional Hierarchy and Reversibility Within the Murine Spermatogenic Stem Cell Compartment. Science 328: 62–67. 10.1126/science.1182868 20299552 PMC2981100

[50] Huch M, Dorrell C, Boj SF, van Es JH, Li VS, et al (2013) In vitro expansion of single Lgr5+ liver stem cells induced by Wnt-driven regeneration. Nature 494: 247–250. 10.1038/nature11826 23354049 PMC3634804

[51] Sandoval-Guzman T, Wang H, Khattak S, Schuez M, Roensch K, et al (2014) Fundamental differences in dedifferentiation and stem cell recruitment during skeletal muscle regeneration in two salamander species. Cell Stem Cell 14: 174–187. 10.1016/j.stem.2013.11.007 24268695

[52] Eiraku M, Takata N, Ishibashi H, Kawada M, Sakakura E, et al (2011) Self-organizing optic-cup morphogenesis in three-dimensional culture. Nature 472: 51–56. 10.1038/nature09941 21475194

[53] Lancaster MA, Renner M, Martin CA, Wenzel D, Bicknell LS, et al (2013) Cerebral organoids model human brain development and microcephaly. Nature 501: 373–379. 10.1038/nature12517 23995685 PMC3817409

[54] Sato T, Clevers H (2013) Growing self-organizing mini-guts from a single intestinal stem cell: mechanism and applications. Science 340: 1190–1194. 10.1126/science.1234852 23744940

[55] Sato T, Vries RG, Snippert HJ, van de Wetering M, Barker N, et al (2009) Single Lgr5 stem cells build crypt-villus structures in vitro without a mesenchymal niche. Nature 459: 262–265. 10.1038/nature07935 19329995

[56] Barker N, Huch M, Kujala P, van de Wetering M, Snippert HJ, et al (2010) Lgr5(+ve) stem cells drive self-renewal in the stomach and build long-lived gastric units in vitro. Cell Stem Cell 6: 25–36. 10.1016/j.stem.2009.11.013 20085740

[57] Yui S, Nakamura T, Sato T, Nemoto Y, Mizutani T, et al (2012) Functional engraftment of colon epithelium expanded in vitro from a single adult Lgr5(+) stem cell. Nat Med 18: 618–623. 10.1038/nm.2695 22406745

[58] Jung P, Sato T, Merlos-Suarez A, Barriga FM, Iglesias M, et al (2011) Isolation and in vitro expansion of human colonic stem cells. Nat Med 17: 1225–1227. 10.1038/nm.2470 21892181

[59] Greggio C, De Franceschi F, Figueiredo-Larsen M, Gobaa S, Ranga A, et al (2013) Artificial three-dimensional niches deconstruct pancreas development in vitro. Development 140: 4452–4462. 10.1242/dev.096628 24130330 PMC4007719

[60] Huch M, Bonfanti P, Boj SF, Sato T, Loomans CJ, et al (2013) Unlimited in vitro expansion of adult bi-potent pancreas progenitors through the Lgr5/R-spondin axis. EMBO J 32: 2708–2721. 10.1038/emboj.2013.204 24045232 PMC3801438

[61] Nanduri LS, Baanstra M, Faber H, Rocchi C, Zwart E, et al (2014) Purification and ex vivo expansion of fully functional salivary gland stem cells. Stem Cell Reports 3: 957–964. 10.1016/j.stemcr.2014.09.015 25448065 PMC4264052

[62] Watt FM (2014) Mammalian skin cell biology: At the interface between laboratory and clinic. Science 346: 937–940. 10.1126/science.1253734 25414300

[63] Karthaus WR, Iaquinta PJ, Drost J, Gracanin A, van Boxtel R, et al (2014) Identification of multipotent luminal progenitor cells in human prostate organoid cultures. Cell 159: 163–175. 10.1016/j.cell.2014.08.017 25201529 PMC4772677

[64] Suga H, Kadoshima T, Minaguchi M, Ohgushi M, Soen M, et al (2011) Self-formation of functional adenohypophysis in three-dimensional culture. Nature 480: 57–62. 10.1038/nature10637 22080957

[65] Meinhardt A, Eberle D, Tazaki A, Ranga A, Niesche M, et al (2014) 3D Reconstitution of the Patterned Neural Tube from Embryonic Stem Cells. Stem Cell Reports 3: 987–999. 10.1016/j.stemcr.2014.09.020 25454634 PMC4264068

[66] Dye BR, Hill DR, Ferguson MA, Tsai YH, Nagy MS, et al (2015) In vitro generation of human pluripotent stem cell derived lung organoids. Elife 4. 10.7554/eLife.05098 PMC4370217 25803487

[67] van den Brink SC, Baillie-Johnson P, Balayo T, Hadjantonakis AK, Nowotschin S, et al (2014) Symmetry breaking, germ layer specification and axial organisation in aggregates of mouse embryonic stem cells. Development 141: 4231–4242. 10.1242/dev.113001 25371360 PMC4302915

[68] Forster R, Chiba K, Schaeffer L, Regalado SG, Lai CS, et al (2014) Human intestinal tissue with adult stem cell properties derived from pluripotent stem cells. Stem Cell Reports 2: 838–852. 10.1016/j.stemcr.2014.05.001 24936470 PMC4050346

[69] Spence JR, Mayhew CN, Rankin SA, Kuhar MF, Vallance JE, et al (2011) Directed differentiation of human pluripotent stem cells into intestinal tissue in vitro. Nature 470: 105–U120. 10.1038/nature09691 21151107 PMC3033971

[70] Watson CL, Mahe MM, Munera J, Howell JC, Sundaram N, et al (2014) An in vivo model of human small intestine using pluripotent stem cells. Nature Medicine 20: 1310–1314. 10.1038/nm.3737 25326803 PMC4408376

[71] Bentley DR, Balasubramanian S, Swerdlow HP, Smith GP, Milton J, et al (2008) Accurate whole human genome sequencing using reversible terminator chemistry. Nature 456: 53–59. 10.1038/nature07517 18987734 PMC2581791

[72] Metzker ML (2010) Sequencing technologies—the next generation. Nat Rev Genet 11: 31–46. 10.1038/nrg2626 19997069

[73] Okita K, Ichisaka T, Yamanaka S (2007) Generation of germline-competent induced pluripotent stem cells. Nature 448: 313–317. 17554338 10.1038/nature05934

[74] Chen BC, Legant WR, Wang K, Shao L, Milkie DE, et al (2014) Lattice light-sheet microscopy: Imaging molecules to embryos at high spatiotemporal resolution. Science 346: 439–439. 10.1126/science.1257998 PMC4336192 25342811

[75] Huisken J, Stainier DYR (2009) Selective plane illumination microscopy techniques in developmental biology. Development 136: 1963–1975. 10.1242/dev.022426 19465594 PMC2685720

[76] Keller PJ, Schmidt AD, Wittbrodt J, Stelzer EHK (2008) Reconstruction of Zebrafish Early Embryonic Development by Scanned Light Sheet Microscopy. Science 322: 1065–1069. 10.1126/science.1162493 18845710

[77] Gould TJ, Burke D, Bewersdorf J, Booth MJ (2012) Adaptive optics enables 3D STED microscopy in aberrating specimens. Opt Express 20: 20998–21009. 10.1364/OE.20.020998 23037223 PMC3635694

[78] Hell SW (2007) Far-field optical nanoscopy. Science 316: 1153–1158. 17525330 10.1126/science.1137395

[79] Huang B, Bates M, Zhuang X (2009) Super-resolution fluorescence microscopy. Annu Rev Biochem 78: 993–1016. 10.1146/annurev.biochem.77.061906.092014 19489737 PMC2835776

[80] Toomre D, Bewersdorf J (2010) A new wave of cellular imaging. Annu Rev Cell Dev Biol 26: 285–314. 10.1146/annurev-cellbio-100109-104048 20929313

[81] Doudna JA, Charpentier E (2014) The new frontier of genome engineering with CRISPR-Cas9. Science 346: 1077-+. 10.1126/science.1258096 25430774

[82] Hsu PD, Lander ES, Zhang F (2014) Development and Applications of CRISPR-Cas9 for Genome Engineering. Cell 157: 1262–1278. 10.1016/j.cell.2014.05.010 24906146 PMC4343198

[83] Shalem O, Sanjana NE, Hartenian E, Shi X, Scott DA, et al (2014) Genome-Scale CRISPR-Cas9 Knockout Screening in Human Cells. Science 343: 84–87. 10.1126/science.1247005 24336571 PMC4089965

[84] Wang T, Wei JJ, Sabatini DM, Lander ES (2014) Genetic Screens in Human Cells Using the CRISPR-Cas9 System. Science 343: 80–84. 10.1126/science.1246981 24336569 PMC3972032

[85] Zhou Y, Zhu S, Cai C, Yuan P, Li C, et al (2014) High-throughput screening of a CRISPR/Cas9 library for functional genomics in human cells. Nature 509: 487–491. 10.1038/nature13166 24717434

[86] Harrison MM, Jenkins BV, O'Connor-Giles KM, Wildonger J (2014) A Crispr View of Development. Genes & Development 28: 1859–1872. 25184674 10.1101/gad.248252.114 PMC4197953

[87] Peng Y, Clark KJ, Campbell JM, Panetta MR, Guo Y, et al (2014) Making designer mutants in model organisms. Development 141: 4042–4054. 10.1242/dev.102186 25336735 PMC4302887

